# Pre-operative diagnosis of an unusual complication of abdominal aortic aneurysm on multidetector computed tomography: a case report

**DOI:** 10.1186/1757-1626-1-231

**Published:** 2008-10-09

**Authors:** George C Jakanani, Peter Lee Chong

**Affiliations:** 1 Department of Radiology, Leicester Royal Infirmary, Infirmary Square, Leicester, LE1 5WW, UK; 2 Department of Vascular Surgery, Lincoln County Hospital, Greetwell Road, Lincoln, LN2 5QY, UK

## Abstract

Spontaneous fistulation of an abdominal aortic aneurysm (AAA) into the inferior vena cava (IVC) is an unusual and infrequently encountered complication in clinical practice. In the majority of cases, it is a diagnosis made on the operating table, during surgical repair of AAA. We report a patient with an aortocaval fistula diagnosed preoperatively on multidetector computed tomography (MDCT). Preoperative diagnosis of this rare complication is important as it allows appropriate anaesthetic and surgical planning thereby reducing morbidity and mortality.

## Case presentation

A 75 year old male was admitted as an emergency with a 12 day history of persisting vague lower abdominal discomfort and constipation. He also reported reduced urine output and lethargy. An ex-smoker, he was on treatment for high blood pressure and high cholesterol.

**Figure 1:**
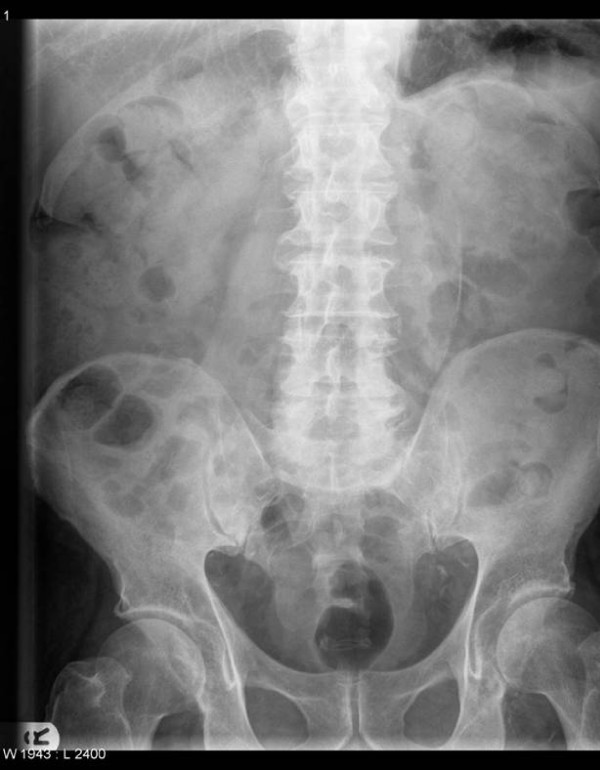
**AXR shows curvilinear calcification in wall of AAA.** The psoas shadow is preserved.

**Figure 2:**
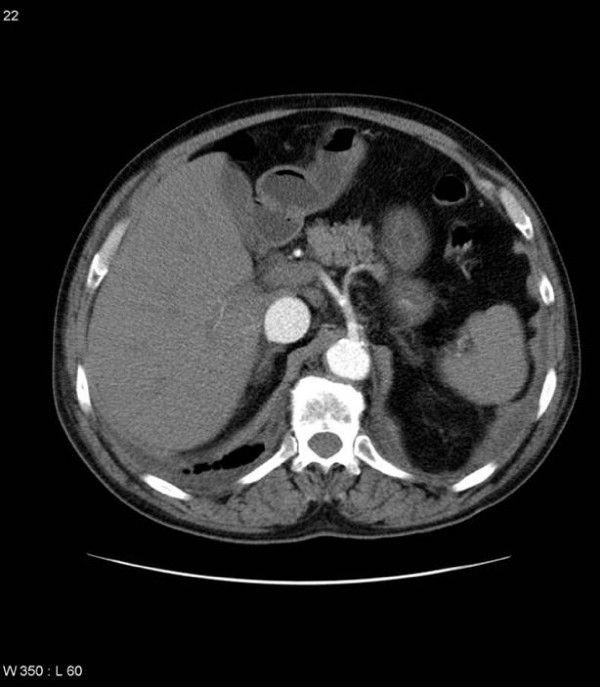
**Selected axial MDCT slice at level of celiac axis.** Note the early opacification of the IVC, which is also engorged.

**Figure 3:**
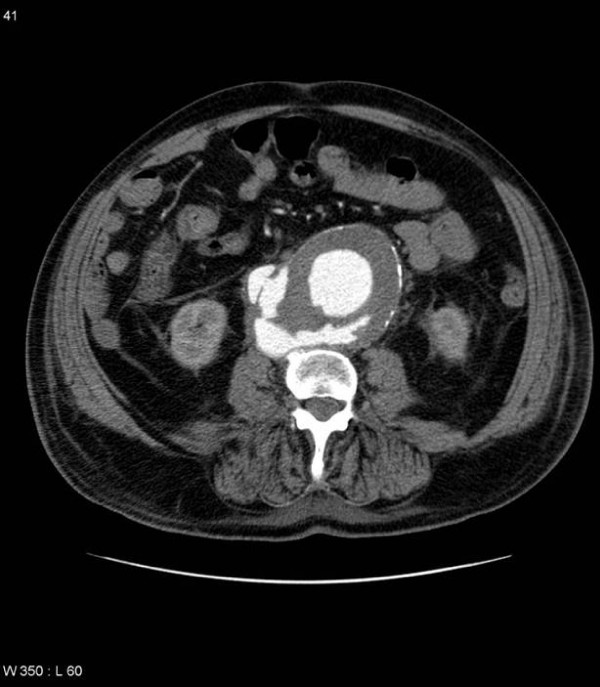
**Selected axial MDCT slice at a lower level shows an infrarenal AAA with fistulous communication with the IVC.** Note the absence of the normal fat plane between the aorta and IVC.

On examination he had a palpable abdominal aneurysm but was haemodynamically stable. Plain abdominal X-ray showed curvilinear wall calcification in a AAA (Fig. 1). A CT scan was performed to exclude a leaking aneurysm and revealed a 7 cm infrarenal abdominal aortic aneurysm with fistulation into the inferior vena cava (Fig. 2 and Fig. 3).

The patient's constipation was treated with symptomatic improvement. During this period, preoperative assessments were performed and the patient scheduled for urgent planned repair of the AAA. However, before the theatre date he developed high output cardiac failure, manifesting as acute shortness of breath with acute pulmonary oedema and lower limb oedema. As a result he underwent emergency open repair of the aneurysm with a tube graft, together with closure of the fistula. He made an uneventful recovery and was discharged home 12 days post operatively.

## Discussion

The incidence of spontaneous aortocaval fistulation from AAA is variably reported, from less than 1% to 10% [1,2]. Morbidity and mortality are high, and prompt repair is warranted if the patient is to survive. The classic clinical presentation is of a patient with a tender aneurysm with a palpable thrill and bruit, and warm peripheries [3]. Some patients will report feeling a sensation of fullness, and that the heart is about to burst, the aptly named ''bursting heart '' syndrome [4]. Many will develop high output heart failure along with bilateral lower limb swelling and discolouration due to venous hypertension. [5]

The preoperative diagnosis of aortocaval fistula improves morbidity and mortality by allowing appropriate anaesthetic and surgical planning. It is important for the anaesthetist to administer IV fluids judiciously and to avoid intra-operative fluid overload that would exacerbate pre-existing heart failure. Patients are prone to acute cardiac decompensation particularly at the time of aortic cross-clamping and fistula closure. Pre-operative diagnosis allows the surgeon to plan the approach to repair, and to apply a meticulous technique that amongst other things does not dislodge thrombus into the IVC to cause pulmonary embolism.

In this particular case endovascular placement of a covered stent to exclude the aneurysm was not considered appropriate as this would still leave a fistulous connection between the IVC and the aneurysm sac, the equivalent of a type 2 endoleak.

The signs of an aortocaval fistula on CT are the presence of an AAA, with early and almost equal opacification of the IVC during the arterial phase of contrast enhancement, and engorgement of its proximal portion. What sets MDCT apart from earlier generations of CT, and is so well demonstrated in our patient is that direct visualisation of the fistula is now possible with the fine cuts and consequently excellent resolution that MDCT affords. The additional information provided by multiplanar reconstructions increases the diagnostic utility of MDCT and provides a useful visual map for the operating surgeon.

## Conclusion

With MDCT now widely used in emergency departments in the investigation of suspected leaking AAA, it is important for reporting radiologists and vascular surgeons to be aware of the imaging appearance of this unusual complication, if the benefits of preoperative diagnosis are to be realised.

## Competing interests

The authors declare that they have no competing interests.

## Authors' contributions

GCJ prepared the manuscript. PLC looked after the patient, and edited the manuscript. All authors read and approved the final manuscript.

## Consent

Written informed consent was obtained from the patient for publication of this case report and accompanying images. A copy of the written consent is available for review by the Editor-in-Chief of this journal.
